# Coronary Artery Anomalies and Their Impact on the Feasibility of Percutaneous Pulmonary Valve Implantation

**DOI:** 10.1007/s00246-021-02684-0

**Published:** 2021-08-07

**Authors:** Anja Hanser, Jörg Michel, Andreas Hornung, Ludger Sieverding, Michael Hofbeck

**Affiliations:** Department of Pediatric Cardiology, University Children’s Hospital Tuebingen, Hoppe-Seyler-Str. 1, 72076 Tuebingen, Germany

**Keywords:** Congenital heart disease, Percutaneous pulmonary valve implantation, Congenital coronary artery anomaly

## Abstract

One of the major obstacles preventing successful percutaneous pulmonary valve implantation (PPVI) is related to the close proximity of coronary artery branches to the expected landing zone. The aim of this study was to assess the frequency of coronary artery anomalies (CAAs) especially those associated with major coronary branches crossing the right ventricular outflow tract (RVOT) and to describe their relevance for the feasibility of percutaneous pulmonary valve implantation (PPVI). In our retrospective single-center study 90 patients were evaluated who underwent invasive testing for PPVI in our institution from 1/2010 to 1/2020. CAAs were identified in seven patients (8%) associated with major branches crossing the RVOT due to origin of the left anterior descending (LAD) or a single coronary artery from the right aortic sinus. In 5/7 patients with CAAs balloon testing of the RVOT and selective coronary angiographies revealed a sufficiently large landing zone distal to the coronary artery branch. While unfavorable RVOT dimensions prevented PPVI in one, PPVI was performed successfully in the remaining four patients. The relatively short landing zone required application of the “folded” melody technique in two patients. All patients are doing well (mean follow-up 3 years). CAAs associated with major coronary branches crossing the RVOT can be expected in about 8% of patients who are potential candidates for PPVI. Since the LAD crossed the RVOT below the plane of the pulmonary valve successful distal implantation of the valve was possible in 4/7 patients. Therefore these coronary anomalies should not be considered as primary contraindications for PPVI.

## Introduction

Percutaneous pulmonary valve implantation (PPVI) has found widespread acceptance in the postoperative revalvulation of the dysfunctional right ventricular outflow tract [1–4]. Major limitations for this procedure are coronary artery branches located in close proximity to the expected landing zone of the pulmonary valve resulting in the potential risk for coronary compression caused by radial tension from the balloon-expandable stent. Careful pre-interventional assessment of the coronary artery anatomy is therefore required in all patients selected for this procedure to avoid the catastrophic complication of coronary artery compression [5–7].

Specific attention is required in patients with coronary artery anomalies (CAAs). In patients with origin of the left anterior descending coronary artery (LAD) from the right aortic sinus the LAD crosses the right ventricular outflow tract (RVOT) in close proximity to a potential landing zone. This coronary anomaly is not uncommon in patients with tetralogy of Fallot, who represent a major percentage of potential candidates for PPVI [7–9]. The aim of this study was to assess the frequency and relevance of CAA impacting the feasibility of PPVI based on retrospective evaluation of all patients who underwent invasive testing in our institution.

## Materials and Methods

Included in our retrospective study were all patients who underwent cardiac catheterization and balloon interrogation of the RVOT for possible PPVI in our center from 1/2010 to 1/2020. It was the policy of our institution to offer testing for PPVI to all patients. Patients with known coronary artery anomalies in the presence of a conduit or RVOT of potentially treatable size were not excluded from invasive testing. Except for absence of informed consent of the patients or parents to the procedure there were no exclusion criteria. Prior to cardiac catheterization all patients underwent clinical examination, ECG, echocardiogram and chest X-ray. Data from cardiac MRI or CT were available in 66 and 15 patients, respectively. Invasive testing for PPVI included angiographies into the aortic root during simultaneous balloon inflation in the pulmonary outflow tract. In the majority of cases balloon interrogation was performed with Tyshak^R^ balloon catheters (NuMED Inc., Hopkinton, NY, USA) with a diameter of 25 or 30 mm and a length of 4–6 cm. Due to residual uncertainty regarding the proximity of coronary arteries to the expected landing zone additional selective coronary angiographies were required in 25 patients. In 16 of these selective coronary angiographies were performed with simultaneous balloon inflation in the RVOT. Aortic root angiography in the laid-back technique was introduced in routine testing in 2013. We reviewed all angiographies performed in these patients for evaluation of the coronary artery anatomy and right ventricular outflow tract. Special attention was paid to detect anomalies of the coronary artery origin especially those resulting in major branches crossing the right ventricular outflow tract and their impact on the feasibility of PPVI. Unfavorable coronary artery anatomy was defined as either close proximity of a major coronary branch to the expected landing zone (especially in the context of restricted coronary artery mobility during balloon inflation) or compression and impeded flow in the coronary artery in the presence of the inflated balloon. Assessment of coronary artery anatomy included description of variants of origin and the site of close proximity or compression during balloon interrogation of the RVOT.

Statistical analysis was conducted using SPSS, version 27 (IBM Corp., Armonk, NY). This retrospective study was approved by the ethical committee of the University of Tuebingen (project number 307/2020BO2).

## Results

Ninety patients underwent evaluation for possible PPVI. Mean age at the time of invasive testing was 22.8 years (median age 20.3 years, age range 6.9–67.7 years, SD 12.4 years). Mean weight was 57.8 kg (median weight 57.8 kg, weight range 23–153 kg, SD 23.5 kg). Among our patients 60 were male and 30 were female. All patients had congenital heart disease associated with severely compromised pulmonary valve function (Table 1). At the time of evaluation 56 patients (62%) had undergone previous surgery with implantation of a valved or nonvalved conduit between the subpulmonary ventricle and the pulmonary artery. One of these patients had two conduits from the subpulmonary ventricle to the pulmonary artery, and another patient had a conduit in addition to a stenotic but patent right ventricular outflow tract. Thirty-three patients (37%) had undergone surgery of the pulmonary outflow including pulmonary commissurotomy, pulmonary valvectomy, or transannular patch enlargement. One patient had congenital pulmonary regurgitation without any preceding intervention or surgery.

**Table 1:** Diagnosis and anatomy of the RVOT in 90 patients with invasive PPVI testing

Diagnosis	RVOT treated with homograft/contegra/valveless conduit/stented valve	Conduit-free RVOT following surgery	Native RVOT	Total
ToF	25 (22)	25 (18)		50 (40)
PA-VSD	14 (12)			14 (12)
DORV	3 (3)	2 (0)		5 (3)
TAC	2 (2)			2 (2)
AOST s.p. Ross	5 (4)			5 (4)
PST	1 (1)	1 (0)	1 (1)	3 (2)
Complex TGA	5 (5)			5 (5)
Miscellaneous lesions^a^	1 (1)	5 (3)		6 (4)
Total	56 (50)	33 (21)	1 (1)	90 (72)

The numbers in brackets refer to patients who underwent successful PPVI

*RVOT* right ventricular outflow tract, *ToF* tetralogy of Fallot, *PA-VSD* pulmonary atresia with ventricular septal defect, *DORV* double outlet right ventricle, *TAC* truncus arteriosus communis, *AOST* aortic valve stenosis, *PST* pulmonary valve stenosis, *TGA* transposition of the great arteries, *VSD* ventricular septal defect, *AVSD* atrioventricular septal defect, *PAiVS* pulmonary atresia with intact ventricular septum

^a^Miscellaneous lesions include VSD and AVSD with PST, PAiVS

Seventy-two patients (80%) underwent successful implantation of pulmonary valves with one of three different balloon-expandable devices: Melody™ (Medtronic, Minneapolis, USA), SAPIEN XT, and SAPIEN 3 (both Edwards Lifesciences LLC, Irvine, USA). These included 69 Melody™ valves in 67 patients. Two patients with dual connections of the subpulmonary ventricle to the pulmonary artery were treated by implantation of two Melody™ valves. One of these patients has been published before [10]. Three patients received SAPIEN XT valves and two patients SAPIEN 3 valves. Prestenting was performed in all patients using BIB Balloons (NuMED, Hopkinton, USA). Additional dilatation of the prestents—if required—was performed with Atlas^R^ Gold high-pressure balloons (Bard Inc., Tempe, USA).

The option of PPVI was declined in 18/90 (20%) of patients who underwent invasive testing (Table 2). This was exclusively due to coronary artery proximity or compression in 12 patients, while in another 4 patients an unfavorably large RVOT was associated with close proximity of a coronary artery branch to the fully expanded testing balloon. In the remaining two patients the landing zone exceeded the dimensions for placement of the Melody™ or SAPIEN valves available at that time (Table 2). CAAs were present in three of the 18 patients.

**Table 2:** Patients rejected for PPVI after invasive testing

Patient	Diagnosis	Status RVOT	CP, CC	CAA	RVOT size
1	ToF	Conduit-free RVOT	LMC	No	
2	ToF	Conduit-free RVOT	LMC	No	
3	ToF	Conduit-free RVOT	LAD	No	
4	ToF	Conduit-free RVOT	LAD	No	
5	ToF	Homograft	LMC	No	
6	ToF	Homograft	LMC	No	
7	PA-VSD	Homograft	RCA	No	
8	ToF, absent pulmonary valve	Homograft	LMC	No	
9	DORV	Conduit-free RVOT	LAD	No	
10	AVSD, PST	Conduit-free RVOT	LAD	No	
11	AOST, s.p. Ross	Homograft	LAD	No	
12	ToF	Conduit-free RVOT	LAD	No	Inadequate
13	ToF	Conduit-free RVOT	LAD	No	Inadequate
14	VSD, PST	Conduit-free RVOT	LMC	No	Inadequate
15	PA-VSD	Valveless conduit	LMC	LCA from RCA	
16	DORV	Conduit-free RVOT	–	LAD from RCA	Inadequate
17	PST	Conduit-free RVOT	LAD	LAD from RCA	Inadequate
18	ToF	Conduit-free RVOT	–	no	Inadequate

*RVOT* right ventricular outflow tract, *CP/CC* coronary artery proximity/compression, *CAA* coronary artery anomaly, *ToF* tetralogy of Fallot, *PA-VSD* pulmonary atresia with ventricular septal defect, *DORV* double outlet right ventricle, *VSD* ventricular septal defect, *PST* pulmonary valvular stenosis, *AVSD* atrioventricular septal defect, *AOST* aortic valve stenosis, *LMC* left main coronary, *LAD* left anterior descending coronary artery, *RCA* right coronary artery

**Fig. 1:**
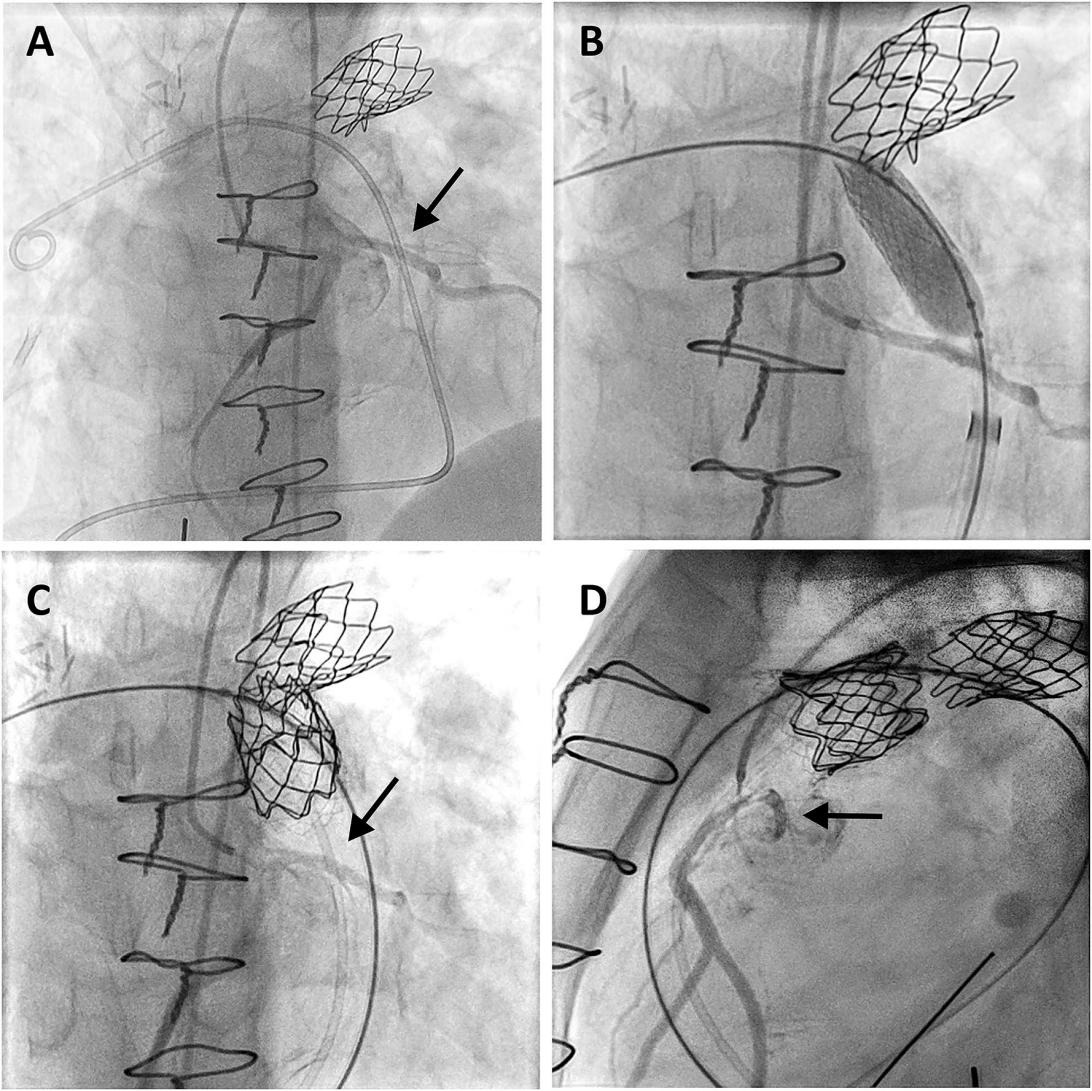
Coronary angiography in pat. 5 (Table 3) shows origin of the RCA and LAD from the right coronary sinus (**A**). Angiography during placement of the stent in the landing zone confirms patency of the LAD (arrows) crossing the RVOT below (**B**). Final coronary angiography following placement of the folded Melody valve shows patent LAD (arrows) proximal to the valve (**C**, **D**)

**Fig. 2:**
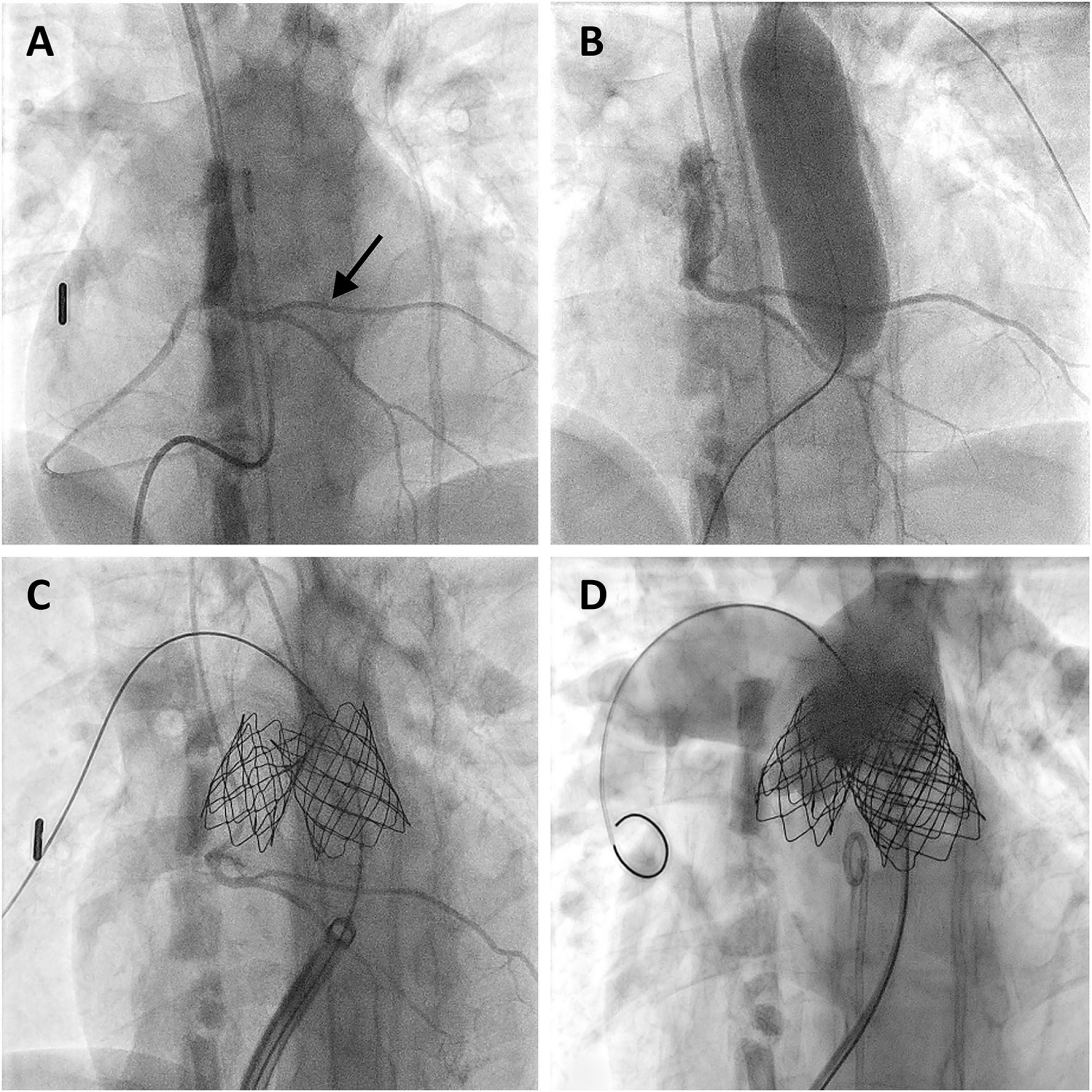
Selective angiography of the right coronary artery in pat. 7 (Table 3) shows origin of the LAD (arrows) from the right coronary sinus crossing the RVOT (**A**). Selective angiography during balloon testing of the RVOT reveals that the LAD is located well below the expected landing zones both in the RVOT and in the PTFE conduit (**B**). Confirmation of coronary patency following placement of a Melody valve in the conduit and creation of a landing zone in the RVOT (**C**). Final pulmonary angiography shows good function of both valves

Seven patients (8%) with anomalous origin of a major coronary artery branch were encountered in the entire cohort (Table 3). The diagnoses of these patients included tetralogy of Fallot, pulmonary atresia and ventricular septal defect (PA-VSD), double outlet right ventricle (DORV), and pulmonary valvular stenosis. All these patients underwent selective angiographies of both coronary arteries. Anomalous origin of the LAD from the right coronary artery or right aortic sinus crossing the RVOT (Figs. 1, 2) was found in 6 patients. One additional patient had a single coronary artery originating from the right aortic sinus. In this patient the left coronary artery crossed posterior to the RVOT and underneath the pulmonary artery.

**Table 3:** Patients with anomalous origin of a major coronary artery branch

Patient	Diagnosis	Status RVOT	CAA	PPVI
1	PA-VSD	Valveless conduit	LCA from RCA	PPVI aborted, CP/CC
2	PST	Conduit-free native RVOT	LAD from RCA	PPVI aborted, inadequate RVOT, and CP
3	DORV	Conduit-free RVOT	LAD from RCA	PPVI aborted, inadequate RVOT
4	ToF	Contegra-conduit	LAD from RCA	Folded Melody™
5	PA-VSD	Homograft	LAD from RCA	Folded Melody™
6	ToF	Conduit-free RVOT	LAD from RCA	Edwards Sapien3 26 mm
7	DORV	Valveless conduit and native RVOT	LAD from RCA	Melody™ both in conduit and in RVOT

*RVOT* right ventricular outflow tract, *CAA* coronary artery anomaly, *PA-VSD* pulmonary atresia with ventricular septal defect, *DORV* double outlet right ventricle, *PST* pulmonary valve stenosis, *ToF* tetralogy of Fallot, *LCA* left anterior descending coronary artery, *RCA* right coronary artery, *LAD* left anterior descending coronary artery, *CP/CC* coronary artery proximity/compression

In three of these patients the decision was made not to attempt PPVI. In the patient with a single coronary artery the unfavorable course of the left coronary artery underneath the RVOT in close proximity to the expected landing zone prevented PPVI. In two patients with LAD originating from the right aortic sinus PPVI was not attempted due to stretched diameters of the intended landing zone of 31 mm and 28 mm, respectively, and absent indentation of the fully inflated balloon. Since the LAD crossed the outflow tract proximal to the expected position of an implanted valve, PPVI would have been theoretically possible in one of them. In the remaining patients the LAD crossed the RVOT at the level of the expected landing zone in close proximity to the fully expanded balloon (Table 3). In summary among 84 patients with adequate size conduits or RVOT only one patient was excluded from PPVI based on coronary artery proximity/compression due to a CAA.

PPVI was performed without complication in the remaining 4 patients with CAA (mean age = 26.8 years, age range 17.4–32 years). The anatomy of the RVOT included s/p implantation of 18 mm bovine internal jugular vein conduit (Contegra), s/p 22 mm homograft, and conduit-free RVOT following pulmonary valvotomy. The fourth patient had a valveless 18 mm PTFE RV-PA conduit in addition to the patent native RVOT.

Careful analysis of the angiograms in these patients revealed sufficient space for a safe landing zone distal to the LAD crossing the RVOT. All our patients underwent prestenting. In two patients a short landing zone proximal to the bifurcation limited prestenting to the implantation of a 8Z 22 mm CP Stent™ (NuMED Inc, Hopkinton, USA) and a 26 mm LD Max™ stent (ev3 Inc., Plymouth, USA), respectively (Table 3). A shortened Melody™ valve, achieved by folding of the proximal and distal struts, was implanted in both of these patients (Fig. 1). One of these patients and the applied technique have been published before [11]. The third patient was treated by implantation of a 26 mm SAPIEN 3 valve following prestenting with a LD Max™ stent. The fourth patient underwent implantation of Melody™ valves both in the 18 mm PTFE conduit and into the native RVOT following prestenting with 26 mm LD Max™ and 8Z 45 mm covered CP stents, respectively (Fig. 2). In all patients selective angiographies following prestenting and after valve implantation revealed unimpeded flow to the coronary arteries (Figs. 1, 2). The postinterventional course was uneventful and all patients are doing well with a mean follow-up of 3 years.

## Discussion

Following repair of congenital heart defects a significant number of patients are left with long-term impairment of pulmonary valve function. To avoid the sequelae of long-term pulmonary regurgitation and/or residual pulmonary stenosis on right ventricular function PPVI has become the preferred treatment option for many of these patients [2–4]. Excellent results have been reported both with the Melody™ and with the SAPIEN valves [4, 12, 13]. According to the data based on 845 patients from the post-approval MELODY Registry, this treatment option is associated with a low risk of major complications (4.2%) and a low incidence of procedure-related deaths (0.5%) [4]. Recent data showed that PPVI with the Melody™ valve is offering excellent medium- and long-term results comparable to surgical valve replacement [14].

Proximity of a major coronary artery branch to the intended landing zone represents one of the main obstacles for successful PPVI [5, 7]. The option of PPVI treatment was declined in 18/90 (20%) of patients who underwent invasive testing. In 12 patients (13%) this was due to possible interference with a coronary artery branch. In an additional 4 patients with RVOTs exceeding the size of presently available PPVI devices balloon interrogation revealed close proximity of a coronary artery branch to the expected landing zone. However, it should be noted that our cohort may be associated with some bias since some patients with unfavorably large RVOT diameters were referred for surgical intervention without invasive testing of the RVOT based on MRI findings (see also “Limitations of the Study”).

Of specific interest are coronary artery anomalies associated with major coronary branches crossing the RVOT in close proximity to the potential landing zone [5, 7, 15]. Anatomic variations include major coronary artery branches originating either from the right or from the left aortic sinus. Seven patients (8%) with coronary artery anomalies were identified in our study. The most frequent was anomalous origin of the LAD from the right coronary artery or right aortic sinus (six patients) while one patient presented with a single coronary artery originating from the right aortic sinus. Six of these patients had conotruncal malformations including tetralogy of Fallot, PA-VSD as well as DORV (Table 2). Our findings correspond with previous data describing coronary artery anomalies in patients with tetralogy of Fallot. Among 943 surgical patients with tetralogy of Fallot Pontailler et al. found 76 (8%) with anomalous coronary arteries crossing the right ventricular infundibulum [8]. The most frequent anomalies were origin of the main or an accessory LAD from the right aortic sinus accounting for 62% of these anomalies. Major infundibular branches were present in about 20%. Abnormal origin of the right coronary artery from the left aortic sinus or from the LAD was described in 12% while the remaining 6% represented patients with a single coronary originating either from the right or left aortic sinus [8]. Quite similar distributions of anatomic variants were reported by Ruzmetov et al. among 43 patients with tetralogy of Fallot and anomalous coronary arteries [9]. While these coronary anomalies no longer represent a significant risk factor for corrective surgery which frequently can be performed without the use of a right ventricular to pulmonary artery conduit, they remain important for the planning of later repeat surgery or PPVI [8, 9]. Abnormal coronary artery anatomy was demonstrated in 34/226 (15%) patients with tetralogy of Fallot who underwent catheterization and testing for intended PPVI [7]. In this and in other studies coronary artery anomalies were associated with an increased risk of coronary artery compression during balloon interrogation of the RVOT [5, 7, 16]. Despite this statistically increased risk the results of our study show that successful PPVI is possible in the majority of patients exhibiting a major coronary artery branch crossing the RVOT. The presence of these coronary artery anomalies should not be considered as a primary contraindication for PPVI.

In two of our patients with coronary artery anomaly and in 21/90 patients of our entire cohort successful valve implantation was achieved in conduit-free outflow tracts (Table 1). This confirms the experience of recent studies that percutaneous pulmonary valve replacement can be performed with low morbidity in patients with conduit-free RVOTs following pulmonary valvotomy, partial or complete pulmonary valvectomy, patch enlargement, or a combination of these [17–20]. New generations of self-expandable valve prostheses will soon become available for large RVOTs [3]. However, in patients with dysfunctional non-conduit RVOTs and requirement of large valve prostheses aortic root compression with the risk of aortic regurgitation or erosion of the aortic wall may represent a limiting factor [21].

Although the majority of the patients in our study had preceding 3D imaging for evaluation of the anatomy of the RVOT and coronary arteries the final decision for valve implantation was based on the results of angiographic imaging of the RVOT, balloon interrogation of the landing zone, and simultaneous imaging of the coronary arteries. Based on continuous improvements of 3D imaging techniques and introduction of image fusion techniques it can be expected that evaluation of the landing zone and its proximity to coronary artery branches will become even more accurate providing the possibility of exact pre-interventional planning and optimal deployment of the valve [16, 22–28].

## Limitations of the Study

Limitations of our study are due to its retrospective nature and the relatively small sample size. Both the technique of balloon interrogation and the technique of coronary artery imaging experienced improvement during the elapsed period of the study. With regard to the proximity of major coronary artery branches to the expected landing zone we followed a rather conservative approach with the primary goal to minimize the risk of coronary artery compression. This may have reduced the rate of successful implantations but none of our patients experienced problems of coronary compression. Finally, we cannot exclude some selection bias of our cohort. While we did not reject patients because of anatomic variants of coronary artery origin including those associated with major branches crossing the RVOT, some patients with large RVOT beyond the range of percutaneous revalvulation underwent surgery without invasive testing of coronary artery proximity to the RVOT.

## Conclusion

Coronary artery anomalies associated with a major coronary branch crossing the RVOT can be expected in about 8% of patients evaluated for PPVI. In our experience, these coronary anomalies frequently do not represent a contraindication for PPVI. Balloon testing revealed that the LAD frequently crosses the RVOT below the plane of the pulmonary valve and proximal to a potential landing zone. Since the landing zone may be rather short it may require implantation of a low profile pulmonary valve or application of the folded melody technique.

## Data Availability

The datasets used and/or analyzed during the current study are available from the corresponding author on reasonable request.

## Abbreviations

CAA: Coronary artery anomaly
CC: Coronary compression
CMR: Cardiac magnetic resonance
DORV: Double outlet right ventricle
ECG: Electrocardiogram
LAD: Left anterior descending coronary artery
LMC: Left main coronary
PA-VSD: Pulmonary atresia with ventricular septal defect
PPVI: Percutaneous pulmonary valve implantation
PTFE RV-PA conduit: Polytetrafluoroethylene conduit from right ventricle to pulmonary artery
RCA: Right coronary artery
RVOT: Right ventricular outflow tract
ToF: Tetralogy of Fallot

## Code Availability

Data analysis was performed using SPSS; V. 25.0 Corp., NY, USA.
